# *Annona muricata* leaves induced apoptosis in A549 cells through mitochondrial-mediated pathway and involvement of NF-κB

**DOI:** 10.1186/1472-6882-14-299

**Published:** 2014-08-15

**Authors:** Soheil Zorofchian Moghadamtousi, Habsah Abdul Kadir, Mohammadjavad Paydar, Elham Rouhollahi, Hamed Karimian

**Affiliations:** Biomolecular Research Group, Biochemistry Program, Institute of Biological Sciences, Faculty of Science, University of Malaya, Kuala Lumpur, Malaysia; Department of Pharmacology, Faculty of Medicine, University of Malaya, Kuala Lumpur, Malaysia; Department of Pharmacy, Faculty of Medicine, University of Malaya, Kuala Lumpur, Malaysia

**Keywords:** Annona muricata, Lung cancer, Apoptosis, Caspase, Mitochondria, NF-κB

## Abstract

**Background:**

*Annona muricata* leaves have been reported to have antiproliferative effects against various cancer cell lines. However, the detailed mechanism has yet to be defined. The current study was designed to evaluate the molecular mechanisms of *A. muricata* leaves ethyl acetate extract (AMEAE) against lung cancer A549 cells.

**Methods:**

The effect of AMEAE on cell proliferation of different cell lines was analyzed by MTT assay. High content screening (HCS) was applied to investigate the suppression of NF-κB translocation, cell membrane permeability, mitochondrial membrane potential (MMP) and cytochrome *c* translocation from mitochondria to cytosol. Reactive oxygen species (ROS) formation, lactate dehydrogenase (LDH) release and activation of caspase-3/7, -8 and -9 were measured while treatment. The western blot analysis also carried out to determine the protein expression of cleaved caspase-3 and -9. Flow cytometry analysis was used to determine the cell cycle distribution and phosphatidylserine externalization. Quantitative PCR analysis was performed to measure the gene expression of Bax and Bcl-2 proteins.

**Results:**

Cell viability analysis revealed the selective cytotoxic effect of AMEAE towards lung cancer cells, A549, with an IC_50_ value of 5.09 ± 0.41 μg/mL after 72 h of treatment. Significant LDH leakage and phosphatidylserine externalization were observed in AMEAE treated cells by fluorescence analysis. Treatment of A549 cells with AMEAE significantly elevated ROS formation, followed by attenuation of MMP via upregulation of Bax and downregulation of Bcl-2, accompanied by cytochrome *c* release to the cytosol. The incubation of A549 cells with superoxide dismutase and catalase significantly attenuated the cytotoxicity caused by AMEAE, indicating that intracellular ROS plays a pivotal role in cell death. The released cytochrome *c* triggered the activation of caspase-9 followed by caspase-3. In addition, AMEAE-induced apoptosis was accompanied by cell cycle arrest at G_0_/G_1_ phase. Moreover, AMEAE suppressed the induced translocation of NF-κB from cytoplasm to nucleus.

**Conclusions:**

Our data showed for the first time that the ethyl acetate extract of *Annona muricata* inhibited the proliferation of A549 cells, leading to cell cycle arrest and programmed cell death through activation of the mitochondrial-mediated signaling pathway with the involvement of the NF-kB signalling pathway.

## Background

Lung cancer as one of the critical causes of cancer death throughout the world has the prevalent complication of apoptosis resistance against different anticancer agents [1]. Due to the typical asymptomatic progression of lung cancer at an early stage, it is normally diagnosed at an advanced stage (56%). In spite of all the development in chemoradiation and surgical techniques, the 5-year survival rate for patients with advanced stage disease is still 3.6% [2]. In addition, numerous lung cancer survivors suffer from lung dysfunction, particularly patients with the lung surgical history [3]. Thereby, continued research into the development of safe and efficient new anticancer agents against lung cancer cells is urgently necessary for further improvements in cancer therapy.

Apoptosis is a critical physiological process responsible for the homeostatic mechanism and maintenance of cell populations in tissues [4]. Due to the close correlation between the mechanism of apoptosis and the effect of anticancer agents, extensive research has been done on this mode of cell death [5]. The accumulation of reactive oxygen species (ROS) in cancer cells is a critical factor for the induction of apoptosis by natural products [6, 7], since it will result in oxidative DNA damage following by a collapse in mitochondrial membrane potential (MMP) and leakage of cytochrome *c,* which lead to the activation of the caspase cascade [8]. Furthermore, the perturbation in the expression level of Bax and Blc-2 proteins is an important factor to determine the susceptibility of tumor cells to anticancer agents [9]. Previous anticancer studies also proved that constitutive activation of the ubiquitous transcription factor of NF-κB (nuclear factor-kappa B) is involved in governing the promoting tumor progression of solid and hemopoietic malignancies [10, 11]. Therefore, anticancer agents with the ability to suppress the NF-κB translocation are effectively induce the apoptosis in cancer cells.

*Annona muricata* L. known as gravel, guanabana and soursop is a member of Custard-Apple plants in the Annonaceae family due to a custard-like texture of its fruit. It is a small deciduous tree with a height of 5–8 m and roundish canopy [12]. This popular fruit tree has been widely cultivated in many tropical countries and traditionally used for an array of diseases and ailments [13]. Previous studies demonstrated a significant cytotoxicity for *A. muricata* leaves against various cancer cell lines without affecting the normal cells [14, 15]. Due to this tremendous antiproliferative effect, *A. muricata* was described as “the cancer killer” [15]. Ethanolic extract of *A. muricata* leaves was suggested to have apoptosis-inducing potential against myelogenous leukemic K562 cells, although the detailed mechanism of action has not been explained [16]. Amongst constituents isolated from *A. muricata* leaves, namely annonaceous acetogenins, alkaloids and essential oils, annonaceous acetogenins are strongly implied to be responsible for the promising anticancer effect [17]. The principle objective of this study was to examine how *A. muricata* leaves affecting A549 lung cancer cells, and to investigate the possible mechanism of action involved in this effect.

## Methods

### Plant material and extraction procedures

The plant species (*Annona muricata*) collected from Ipoh, Malaysia, was authenticated by Dr. Yong Kien Thai, an ethnobotanist from the department of Biological Sciences, University of Malaya. The voucher specimen number for this plant is KLU47978. The air-dried leaves of *A. muricata* (1 kg) were cut into fine pieces using a mill grinder and soaked in n-hexane (1500 mL, three times) in conical flasks for four days at room temperature (25–27°C). The n-hexane extract was filtered and the residues were sequentially re-extracted with ethyl acetate (1500 ml, three times) and methanol (1500 ml, three times) using the same method. The resultant filtrate was concentrated to dryness by a Buchi R110 Rotavapor (Buchi Labortechnik AG, Flawil, Switzerland) at 40°C and stored at – 30°C until use. The isolated extracts were dissolved in dimethyl sulfoxide (DMSO) for further experiments.

### Cell culture and MTT assay

MCF-7 (human breast cancer cells), MDA-MB-231 (human breast cancer cells), A549 (human lung cancer cells), HepG2 (human hepatoma cells) and WRL-68 (human hepatic cells) cell lines were obtained from American Type Cell Collection (ATCC, Manassas, VA, USA). Cells were cultured in RPMI-1640 medium (Sigma, St. Louis, MO, USA) supplemented with 10% FBS (PAA, Pasching, Austria), 100 U/mL penicillin (PAA) and 50 μg/mL amphotericin B (PAA) at 37°C with 5% CO_2_. The negative control for all the assays was represented by the untreated medium containing vehicle DMSO (0.1%).

The cytotoxicity of the extracts was determined using the MTT assay as originally described by Mossman [18]. Briefly, cells were treated with different concentrations (1.56, 3.12, 6.25, 12.5, 25, 50 and 100 μg/mL) of three isolated extracts (hexane, ethyl acetate and methanol) and curcumin (positive control) in 96-well plates and incubated for 72 h. After the incubation time, MTT dye (20 μL, 5 mg/mL, Sigma) was added to the cells for 4 h followed by incubation with DMSO for 10 min. The colorimetric assay was measured at the absorbance of 570 nm using a microplate reader (Asys UVM340, Eugendorf, Austria). The antiproliferative potential of the extracts was expressed as IC_50_ values. As an ethyl acetate extract of the leaves (AMEAE) demonstrated the lowest IC_50_ value against lung cancer A549 cells, we used only AMEAE to continue this study against A549 cells.

### LDH release assay

To confirm the cytotoxicity of AMEAE, we carried out lactate dehydrogenase (LDH) release assay using Pierce™ LDH Cytotoxicity Assay Kit (Thermo Scientific™, Pittsburgh, PA, USA). In brief, A549 cells were treated with AMEAE at different concentrations for 48 h. The supernatant of treated A549 cells was transferred into 96-well plate to assess the LDH activity. Triton X-100 (2%) served as a positive control was used to completely lyse the cells and release the maximum LDH. Next, the LDH reaction solution (100 μl) was added to the cells for 30 min. The red color intensity presenting the LDH activity was measured by the absorbance at 490 using a Tecan Infinite^®^200 Pro (Tecan, Männedorf, Switzerland) microplate reader. The level of released LDH from treated cells was expressed as a percentage of positive control.

### Acridine orange/propidium iodide (AO/PI) double staining assay

Morphological changes induced by AMEAE in A549 cells were analyzed using Acridine orange/Propidium iodide (AO/PI) double staining assay. Briefly, A549 cells were seeded in 60 mm^2^ culture dishes followed by treatment with AMEAE (10 μg/mL) for 24, 48 and 72 h. After the incubation time, extract-untreated and treated A549 cells were harvested and washed with PBS. Then, the pellets were stained with 10 μg/mL of AO/PI (1 mg/mL). The stained cells were then observed under a BX51 UV-fluorescent microscope (Olympus, Tokyo, Japan) within 30 min.

### Annexin-V-FITC assay

Induction of the early and late apoptosis by AMEAE was further studied via Annexin-V/PI staining assay. Briefly, A549 cells (1 × 10^6^) were plated in 60 mm^2^ culture dishes and treated with vehicle DMSO and AMEAE (10 μg/mL) for 24, 48 and 72 h. After harvest of adherent and suspension cells and washing them twice with PBS, they were re-suspended in Annexin-V binding buffer (BD Biosciences, San Jose, CA, USA) and stained with Annexin-V-FITC (BD) and PI (Sigma) according to the manufacturer’s instructions. The fluorescence intensity of A549 cells was then analyzed by flow cytometry (BD FACSCanto™ II, San Jose, CA, USA) through quadrant statistics for necrotic and apoptotic cell populations. PI was used for detection of the late apoptosis and necrosis while Annexin-V was consumed for the detection of the early and late apoptosis.

### Cell cycle assay

A flow cytometry analysis was carried out to determine the cell cycle distribution in treated A549 cells with AMEAE. In brief, A549 cells (5 × 10^4^ cells/mL) were treated with AMEAE (10 μg/mL) for 24, 48 and 72 h. After fixation with cold ethanol, cells were washed with PBS and stained with PI (50 μl, 10 mg/mL) for 1 h at 37°C. In addition, RNase A (10 mg/mL) was also used to limit the ability of the PI to bind only to DNA molecules. The stained cells were analyzed for DNA content using flow cytometer (BD FACSCanto™ II).

### Reactive oxygen species (ROS) assay

The effect of AMEAE on the ROS formation in A549 cells was determined by ROS assay. Briefly, treated lung cancer cells with AMEAE at different concentrations in 96-well plates were incubated for 24 h. After the incubation time, the treated cells were stained with dihydroethidium (DEH) at 2.5 μg/mL and Hoechst 33342 (500 nM) dyes for 30 min. Then, cells were fixed with paraformaldehyde (3.5%) for 15 min and washed with PBS twice. The Cellomics ArrayScan HCS reader was used to measure the ROS generation in treated A549 cells.

To further determine the role of ROS generation in AMEAE-induced antiproliferative effect, A549 cells were treated with antioxidants prior to treatment with AMEAE and the cell viability was measured after 24 h. In brief, A549 cells in the exponential phase of growth were supplemented with antioxidants superoxide dismutase (SOD, 300 U/mL) and catalase (400 U/mL) for 1 h prior to AMEAE (20 μg/mL) treatment for 24 h. After incubation time, the cell viability analysis was carried out using a microplate reader (Asys UVM340, Eugendorf, Austria).

### Multiple cytotoxicity assay

To simultaneously determine the crucial apoptotic events in A549 cells after treatment with AMEAE, we used Cellomics Multiparameter Cytotoxicity 3 Kit (Thermo Scientific™, Pittsburgh, PA, USA). Briefly, lung cancer A549 cells were seeded into 96-well plates for 24 h. The cells were treated with AMEAE at different concentrations prior to staining the cells with cell permeability and mitochondrial membrane potential (MMP) dyes. Then, cells were fixed and blocked with 1X blocking buffer according to the manufacture’s protocol. Next, primary cytochrome *c* antibody and secondary DyLight 649 conjugated goat antimouse IgG were added for 1 h. Nuclei of treated cells were also stained with Hoechst 33342 dye. Stained A549 cells in 96-well plates were analyzed using ArrayScan high content screening (HCS) system.

### Bioluminescent assays for caspase-8, -9 and -3/7 activities

A dose-dependent study on the caspase-8, -9 and -3/7 activation was carried out using Caspase-Glo^®^ 3/7, 8 and 9 kit (Promega, Madison, WI, USA). In brief, a total of 5 × 10^3^ A549 cells were seeded per well in a white 96-well microplate and incubated with different concentrations of AMEAE for 24 h. Then, caspase-Glo reagent (100 μl) was added to the cells for 30 min. The induced activation of caspases was measured using a Tecan Infinite^®^200 Pro (Tecan, Männedorf, Switzerland) microplate reader.

To determine the protein expression of cleaved caspase-3 and -9, western blot analysis was carried out as previously described in detail [19]. In brief, A549 cells treated with vehicle DMSO or AMEAE at different concentrations were washed with PBS and lysed in ice-cold Radio Immuno Precipitation Assay (RIPA) buffer. Cell extracts (80 μg protein) were subjected to sodium dodecyl sulfate–polyacrylamide gel electrophoresis (SDS–PAGE), transferred to nitrocellulose membrane, probed with anti-β-actin, anti-cleaved caspase-9 and anti-cleaved caspase-3 (Cell Signaling Technology, Danvers, MA, USA). HRP-conjugated secondary antibodies were used followed by the detection of protein expression using the ECL plus chemiluminescence kit (Amersham Biosciences, Piscataway, NJ, USA).

### Quantitative PCR analysis

The expression of the Bax and Bcl-2 in treated A549 cells was analyzed by quantitative PCR analysis. After treatment of A549 cells with the AMEAE extract at different concentrations for 24 h, Zymo Research Quick-RNA™ MiniPrep kit (Zymo Research, Freiburg, Germany) was used to isolate total RNAs according to the manufacture’s protocol. Then, High Capacity RNA-to-cDNA™ kit (Applied Biosystems, Foster City, CA, USA) was used to synthesize complementary DNAs. Quantitative PCR was carried out with TaqMan^®^ Gene Expression Assays and TaqMan^®^ Fast Advanced Master Mix using the Applied Biosystems StepOnePlus™ system. GAPDH was used to normalize all data. The IDs for TaqMan^®^ Gene Expression Assays used in this study are GAPDH: Hs02758991_g1, Bcl-2: Hs00608023_m1 and Bax: Hs00180269_m1.

### Measurement of NF-κB activity

The Cellomics ArrayScan HCS system was used to analyze the suppressive effect of AMEAE on the nuclear translocation of NF-κB induced by TNF-α. The experiment was carried out using Cellomics nucleus factor-κB (NF-κB) activation kit (Thermo Scientific) as previously described [20]. In brief, A549 cells (1.0 × 10^4^ cells/well) were treated with AMEAE at different concentrations in a 96-well plate for 3 h. The treated A549 cells were stimulated by TNF-α (1 ng/mL) for 30 min. Then, cells were fixed and stained according to the manufacturer’s protocol and analyzed using Array Scan HCS Reader and Cytoplasm to Nucleus Translocation Bioapplication software.

### Statistical analysis

Data are presented as mean ± SEM of three individual experiments. Statistical analysis was performed with a one-way ANOVA analysis using the Prism statistical software package (GraphPad Software, USA). Differences were considered as being significant at *p < 0.05.

## Results and discussion

### AMEAE inhibited the proliferation of cancer cells

We first examined the cytotoxic effect of three extracts (hexane, ethyl acetate and methanol) against MCF-7 (human breast cancer cells), MDA-MB-231 (human breast cancer cells), A549 (human lung cancer cells), HepG2 (human hepatoma cells) and WRL-68 (human hepatic cells) cell lines by using MTT assay. Treatment with AMEAE for 72 h significantly reduced the cell proliferation in cancer cells. As shown in Table 1, the results showed the significant cytotoxic activity of ethyl acetate extract towards all cancer cells. The ethyl acetate extract (IC_50_ ranged from 5.09 ± 0.41 to 11.36 ± 0.67 μg/mL) showed the highest cytotoxicity against all cancer cells as compared to others. Furthermore, the ethyl acetate extract exhibited the highest cytotoxic activity towards A549 cells with the IC_50_ of 5.09 ± 0.41 compared to 11.32 ± 1.54 for the positive control curcumin. After 24 and 48 h treatment with AMEAE, A549 cells elicited the IC_50_ values of 17.542 ± 0.92 and 10.612 ± 1.34 μg/mL. It is worth noting that normal human hepatic WRL-68 cells were not noticeably affected by AMEAE treatment. The cell viability results suggested that the AMEAE inhibitory effect is selective for cancer cells.

**Table 1:** **IC**_**50**_ **values of** ***A. muricata*** **leaves extracts on five different cell lines after 72 h treatment**

Extract	IC _50_ (μg/mL)				
	MCF-7	MDA-MB-231	A549	HepG2	WRL-68
Hexane	49.92 ± 2.23	38.72 ± 0.99	21.05 ± 0.42	77.92 ± 2.23	89.53 ± 3.93
Ethyl acetate	6.39 ± 0.43	11.36 ± 0.67	5.09 ± 0.41	9.3 ± 0.91	47.10 ± 1.23
Methanol	85.58 ± 3.55	> 100 *	> 100	> 100	> 100
Curcumin	7.65 ± 0.55	9.34 ± 0.76	11.32 ± 1.54	17.66 ± 1.21	54.24 ± 2.21

The data represent the means ± SEM of three independent experiments.

* > 100 represents the IC_50_ values greater than 100 μg/mL.

### Induction of LDH release by AMEAE

**Figure 1:**
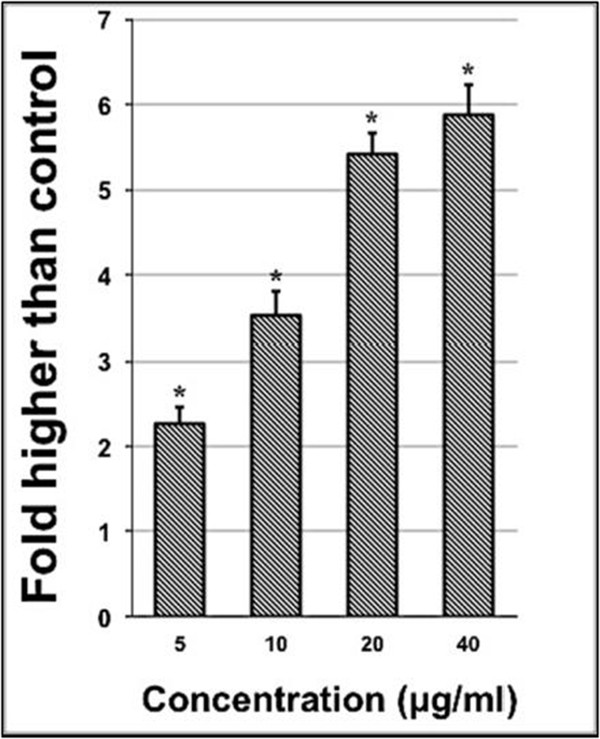
**Lactate dehydrogenase (LDH) assay showed the cytotoxicity of AMEAE against A549 cells.** The result revealed significant cytotoxicity at concentrations of 5 to 40 μg/mL, in a dose-dependent manner. The data represent the means ± SEM of three independent experiments. *p < 0.05 compared with the untreated group.

A stable cytosolic enzyme of lactate dehydrogenase (LDH) catalyzes the oxidation of L-lactate to pyruvate. Upon membrane damage in cells, LDH enzyme is released into the culture medium, suggesting the loss of membrane integrity [21]. Therefore, to further confirm the cytotoxic effect of AMEAE on A549 cells, LDH assay was also performed as another indicator of A549 cytotoxicity. As shown in Figure 1, LDH leakage of A549 cells was significantly increased with the presence of AMEAE by 5.9 folds.

### Quantification of apoptosis using fluorescence microscope and AO/PI double-staining

**Figure 2:**
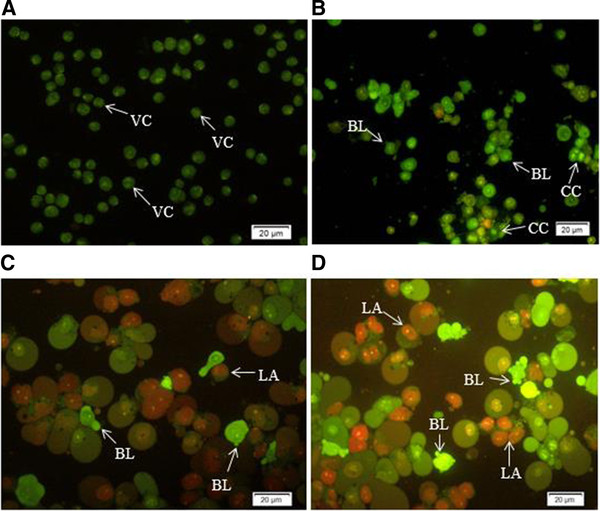
**Fluorescent micrographs of AO/PI-double-stained A549 cells. (A) Untreated A549 cells after 72 h depict healthy structures.** Early apoptosis features such as chromatin condensation and blebbing were observed after **(B)** 24, **(C)** 48 and 72 h **(D)** of treatment with AMEAE. Late apoptosis was observed at the late stage of treatment (magnification: 200×). VC: Viable cells; CC: Chromatin condensation; BL: Blebbing of the cell membrane; LA: Late apoptosis.

Energy-dependent biochemical pathways and distinct morphological features are the main characteristics of apoptosis, or programmed cell death. During the early apoptosis, pyknosis and cell shrinkage are characterized in apoptotic cells [22]. Cell shrinkage is a result of condensation of organelles and the density of cytoplasm, and chromatin condensation or pyknosis is the most critical feature of early apoptosis [23]. The process of late apoptosis is accompanied by budding which contains extensive blebbing of plasma membrane with tightly packed organelles [24]. In the present study, AMEAE was examined for its apoptotic-inducing activity by fluorescence microscopy analysis. Morphological changes in control and treated A549 cells were observed after treatment with AMEAE at 24, 48 and 72 h. It is noteworthy that at 72 h the control cells remained intact and displayed normal structures with a green intact nuclear structure (Figure 2A). After 24 h of treatment, clear signs of apoptosis, such as cytoplasmic shrinkage and membrane blebbing, were observed at a treatment concentration of 10 μg/mL. The early apoptotic cells were detected via the binding of AO within the fragmented DNA emanating a bright green fluorescence. At 24 h treatment with AMEAE, moderate apoptosis was seen by nuclear chromatin condensation and blebbing (Figure 2B). In addition, the late stages of apoptosis as indicated by changes such as the presence of a reddish-orange color because of the binding of PI to denatured DNA were observed after 48 and 72 h of treatment (Figure 2C and D). The results showed that AMEAE generated morphological features that are associated with apoptosis in a time-dependent manner.

### Induction of early and late apoptosis using flow cytometry analysis

**Figure 3:**
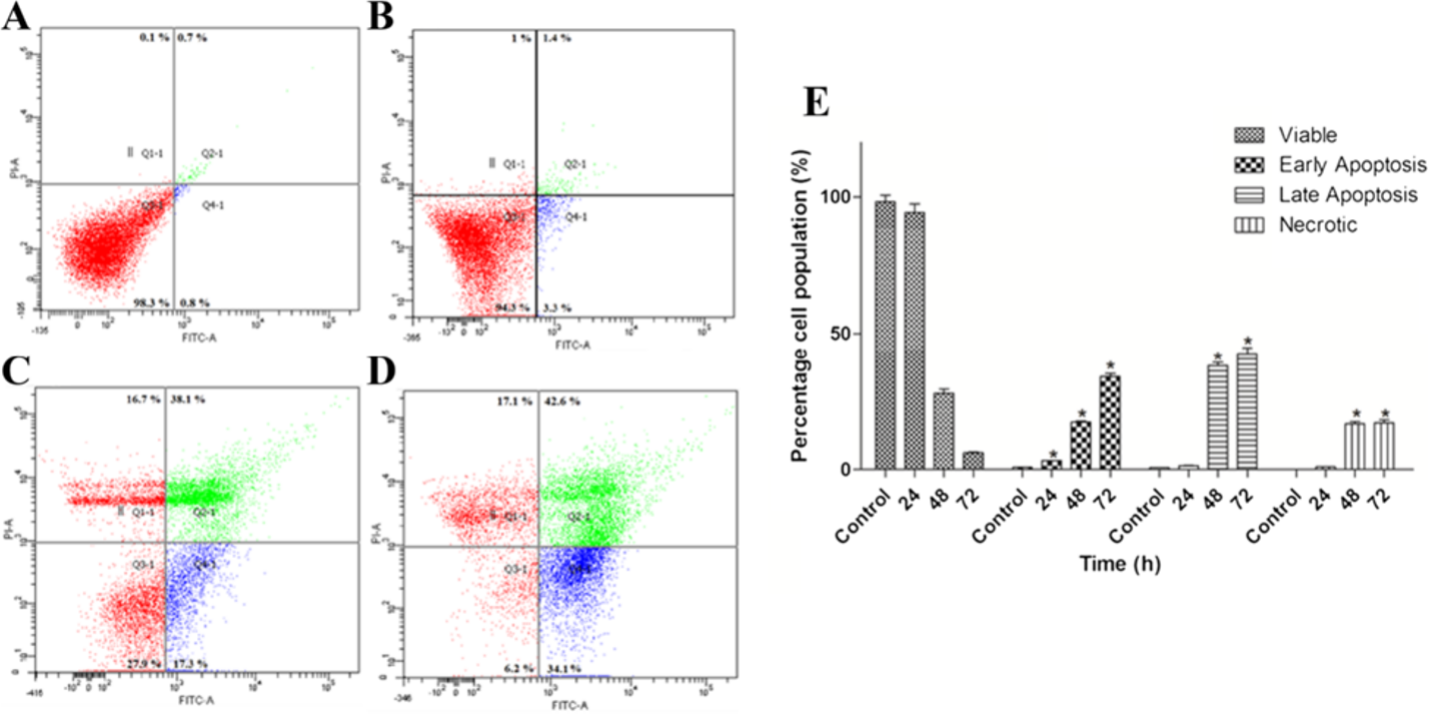
**Time-dependent induction of early and late apoptosis by AMEAE indicated the percentage of early apoptotic, late apoptotic and necrotic cells after (B) 24, (C) 48 and (D) 72 h of treatment. (A)** shows the cells treated with vehicle DMSO. The early and late apoptotic events are depicted in the lower right and upper left quadrant of each panel. **(E)** Representative bar chart demonstrated significant elevation in a number of early and late apoptotic cells after 48 h of treatment. The data represent the means ± SEM of three independent experiments. *p < 0.05 compared with the untreated group.

Our investigations thus far revealed the typical morphological features of apoptosis in AMEAE-treated A549 cells. The morphological changes in apoptotic cells are accompanied by several biochemical modifications, including DNA breakdown, protein cross-linking and protein cleavage which lead to the phagocytic recognition of apoptotic cells by adjacent cells [25]. One such biochemical modification is the expression of cell surface markers such as inward-facing phosphatidylserine which translocates to the outer side of plasma membrane during the early apoptosis [26]. To investigate the biochemical characterization of apoptosis, we examined the externalization of phosphatidylserine (PS) in A549 cells by Annexin-V-FITC assay. Annexin-V as a recombinant phosphatidylserine-binding protein with a high affinity for externalized PS is used for detection of early apoptosis [27]. PI was used for detection of the late apoptosis and necrosis, while Annexin-V was for the detection of the early and late apoptosis. Accordingly, to evaluate whether A549 cells undergo apoptosis, untreated and AMEAE-treated cells were stained with Annexin-V and PI. Flow cytometry analysis of stained cells can distinguish the cells into four categories, namely viable (Annexin-V and PI negative), early apoptosis (Annexin-V positive, PI negative), late apoptosis (Annexin-V and PI positive) and necrotic (Annexin-V negative, PI positive) cells. As illustrated in Figure 3, AMEAE treatment at different time periods (24, 48 and 72 h) resulted in a time-dependent increment of early apoptotic and late apoptotic populations which peaked at 34.1% and 42.6%, respectively, after 72 h treatment.

### AMEAE-induced G_1_ cell cycle arrest

**Figure 4:**
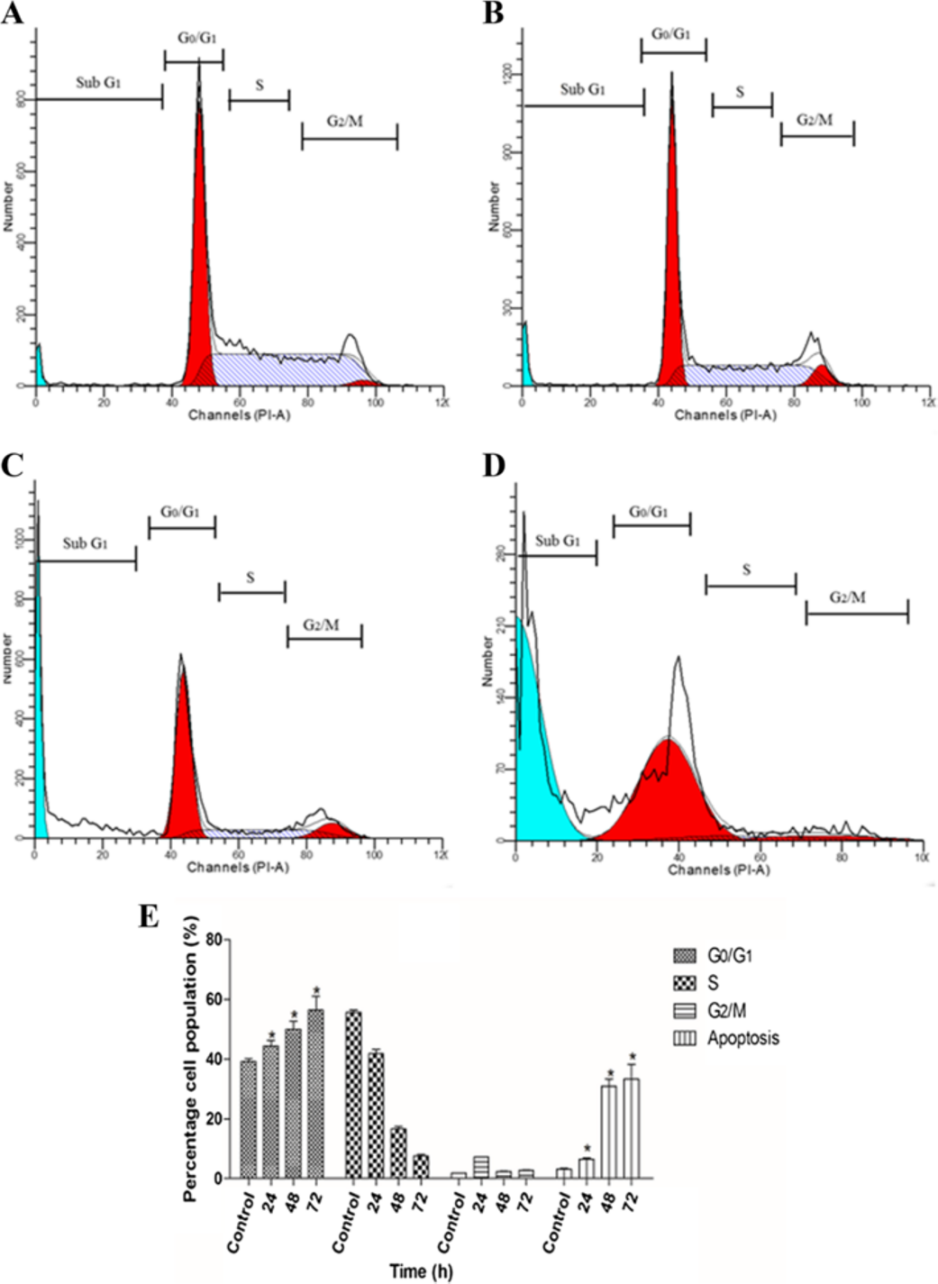
**Flow cytometry analysis on cell cycle progression in A549 cells was carried out after incubation with AMEAE for (A) 0, (B) 24, (C) 48 and (D) 72 h. (E)** Representative bar chart demonstrated significant cell cycle arrest at the G_1_ phase. The data represent the means ± SEM of three independent experiments. *p < 0.05 compared with the no-treatment group.

Cancer is often considered as a disease of cell cycle deregulation. Cell size, extracellular growth signals and DNA integrity are tightly regulated by multiple checkpoints in cell cycle progression [28]. Cancer can originate from perturbation in the expression of positive or negative regulators of cell cycle machinery leading to abnormal proliferation of cancer cells [29]. Thus, induction of cell cycle arrest in cancer cells is considered to be one of the crucial cancer treatment strategies. Phytochemicals with the ability to modulate the cell cycle progression are gaining extensive attention because of the supporting evidences of the concomitant involvement of cell cycle suppression and apoptosis [30, 31]. Thus, flow cytometry analysis was performed to evaluate the effect of AMEAE on the DNA content at various cell cycle checkpoints of A549 cells by cell cycle phase distribution (G_0_, G_1_, S, G_2_ and M) after treatment for 24, 48 and 72 h. As shown in Figure 4, there was a significant G_0_/G_1_ phase arrest in a time-dependent manner demonstrating that AMEAE arrested cell cycle progression at the G_0_/G_1_ phase (Figure 4). Moreover, the increase in the sub-G_1_ phase confirms the apoptosis findings.

### ROS generation induced by AMEAE

**Figure 5:**
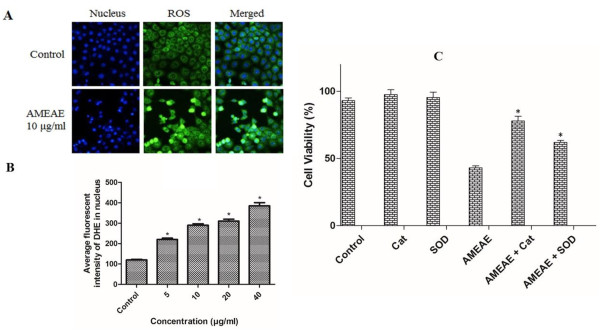
**Effect of AMEAE on ROS generation in A549 cells. (A)** Representative images of A549 cells treated with medium alone and 10 μg/mL of AMEAE for 24 h and stained with dihydroethidium and Hoechst 33342 dyes for 30 min. **(B)** Representative bar chart indicated dose-dependent elevation in ROS formation in treated A549 cells. The level of ROS was significantly elevated at 5 to 40 μg/mL concentrations. **(C)** Pretreatment of A549 cells with Cat and SOD for 1 h prior to the incubation with AMEAE caused a significant elevation in cell viability compared to the treatment alone with AMEAE. The data represent the means ± SEM of three independent experiments. *p < 0.05 compared with the untreated group.

Reactive oxygen species are produced as the result of normal metabolism during the reduction of oxygen to water. Oxidative stress due to increase in the level of intracellular ROS leads to a variety of biochemical and physiological lesions following to metabolic impairments and cell death [32]. The stimulus of free radicals and oxidative stress can trigger the mitochondrial initiated events leading to the activation of intrinsic pathway [33]. Thus, there is an upsurge of interest in cancer studies to use this major role for induction of apoptosis in cancer cells. To investigate this association, the levels of intracellular ROS in A549 cells treated with AMEAE was examined by flow cytometry analysis. In the present study, it was found that AMEAE has noteworthy potential in the induction of ROS generation in A549 cells as shown in Figure 5. As a result of ROS generation, non-fluorescent dihydroethidium (DHE) convert to the fluorescent dye of ethidium, which can bind to DNA. A dose-dependent elevation in ethidium fluorescence in the nucleus was detected in treated A549 cells (Figure 5A). As shown in Figure 5B, ROS production was at the basal level in control untreated A549 cells. In contrast, treatment with AMEAE (24 h) resulted in dose-dependent increase of ROS production as shown by increased ethidium staining in the nucleus (Figure 5A and B). However, pretreatment of A549 cells with antioxidants, namely SOD and Cat significantly increased the cell viability after 24 h compared to the treatment with AMEAE alone (Figure 5C). The viable cells was 62% and 78% by pretreatment with SOD and Cat, respectively, compared with only about 43% by treatment with AMEAE alone. This result supports the close correlation between ROS burst and cytotoxic effect of AMEAE, which suggests to be through activation of the mitonchondrial initiated events.

### Mitochondria-initiated events induced by AMEAE

During apoptosis, mitochondrial membrane potential is frequently disrupted due to the formation of permeability transition pores or the insertions of proapoptotic proteins, such as, Bid or Bax in the mitochondrial membrane [34]. A variety of non-receptor mediated stimuli, including free radicals, radiation, hypoxia and toxin can trigger the intrinsic signaling pathway [26]. The stimuli produce intracellular signals that cause loss in MMP and the opening of the mitochondrial permeability transition pore. These changes in the inner mitochondrial membrane lead to the release of various proteins from the intermembrane space into the cytosol [35]. The main group of released proteins consists of serine protease HtrA2/Omi, cytochrome *c* and Smac/DIABLO. Cytochrome *c* forms apoptosome through binding to pro-caspase-9 as well as Apaf-1. Apoptosome activates caspase-9 following with activation of caspase-3 [36, 37].

**Figure 6:**
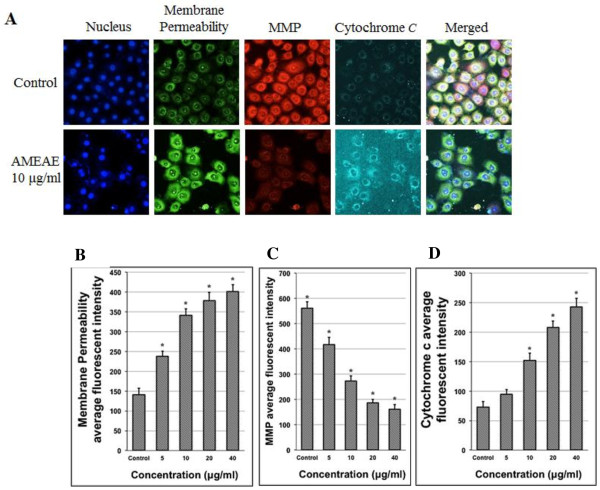
**Effect of AMEAE on cell membrane permeability, MMP and cytochrome** ***c*** **release. (A)** Representative images of A549 cells treated with medium alone and 10 μg/mL of AMEAE, and stained with MMP, cell membrane permeability, Hoechst 33342 and cytochrome *c* dyes. AMEAE caused a marked elevation in cytochrome *c* leakage and cell membrane permeability, and a noteworthy decrease in MMP. Representative bar charts indicating dose-dependent increased **(B)** cell permeability and reduced **(C)** MMP in treated A549 cells at 5 to 40 μg/mL concentrations. Meanwhile, **(D)** cytochrome *c* was significantly released at 10 to 40 μg/mL concentrations. The data represent the means ± SEM of three independent experiments. *p < 0.05 compared with the untreated group.

It is well established that extensive ROS production could result in the disruption of the plasma membrane and mitochondrial damage, thus we further examined the cell membrane permeability, MMP and cytochrome *c* translocation [38]. To determine the function of mitochondria, we applied MMP fluorescent probe. As shown in Figure 6A, untreated A549 cells were strongly stained with red MMP dye, while treated A549 cells with AMEAE were not markedly stained after 24 h. The dose-dependent reduction in fluorescence intensities of MMP was associated with significant elevation in cell membrane permeability which is shown by green fluorescence intensities in Figure 6A. Furthermore, AMEAE significantly elevated the translocation of cytochrome *c* from mitochondria to cytosol in A549 cells after 24 h. In the treated A549 cells, the cyan fluorescence intensities of cytochrome *c* dye (Figure 6A) were markedly increased presenting the significant cytochrome *c* release from mitochondria. The present findings demonstrated that treatment with AMEAE revealed a concentration-dependent increase in membrane permeability, attenuation of MMP, and increase in cytochrome *c* in the cytosol when compared to the control.

### AMEAE induced caspase-8, -9 and -3/7 activation

**Figure 7:**
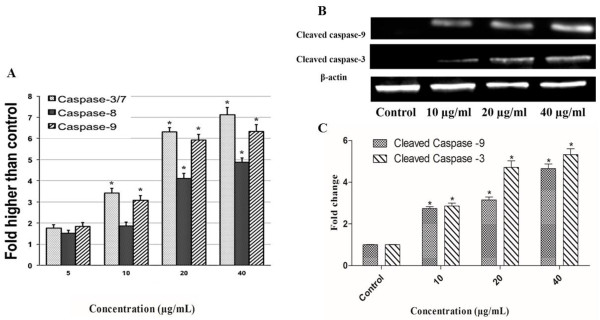
**Effect of AMEAE of caspases activation. (A)** Relative luminescence dose-dependent activation of caspase-8, -9 and -3/7 in A549 cells treated with various concentrations of AMEAE. Caspase-9 and -3/7 were significantly activated at 10 to 40 μg/mL concentrations. Meanwhile, caspase-8 was activated at higher concentrations (20 and 40 μg/mL). **(B)** A549 cells were treated with the indicated concentrations of AMEAE for 24 h. The cell lysates were evaluated for levels of cleaved caspase-3 and -9. AMEAE induced the up-regulation of cleaved caspase-3 and -9. **(C)** The quantitative analysis was expressed as a ratio to the expression of β-actin. The data represent the means ± SEM of three independent experiments. *p < 0.05 compared with the untreated group.

Caspase activation with proteolytic effect at aspartic acid residues are involved in an energy-dependent cascade of molecular events towards apoptosis. To date, two major groups of caspases have been identified to be involved in apoptosis pathways, including executioners or effectors (caspase-3, -6, -7) and initiators (caspase-2, -8, -9, -10) [36]. Intrinsic and extrinsic pathways are activated by their own initiator caspases, namely caspase-9 and -8, respectively. The activation of initiator caspases will, in turn, lead to activation of executioner caspases. The most critical of executioner caspases is considered to be caspase-3 [26]. The execution pathway leads to cytomorphological changes, namely chromatin condensation, cell shrinkage, formation of apoptotic bodies followed by phagocytosis of the apoptotic cell [39]. In the present study, we investigated whether AMEAE can activate the caspases by examining A549 cells treated with different concentrations for 24 h. The activity of both caspase-9 and caspase-3/7 were significantly elevated at 10, 20 and 40 μg/mL AMEAE treatment, while activation of caspase-8 was only triggered at higher concentrations of 20 and 40 μg/mL, suggesting that AMEAE induced apoptosis predominantly through mitochondrial-mediated intrinsic pathway (Figure 7). The western blot analysis of caspases also showed that the protein expression of cleaved caspase-3 and -9 was dose-dependently increased upon treatment with AMEAE. The caspase-3 and -9 protein expressions were significantly up-regulated at 10 to 40 μg/mL concentrations. These results suggest the involvement of caspase cascade in AMEAE-mediated apoptosis.

### AMEAE induced upregulation of Bax and downregulation of Bcl-2 at the gene expression level

**Figure 8:**
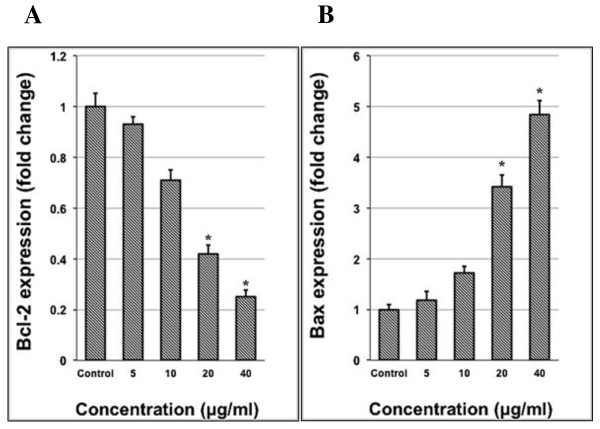
**Effect of AMEAE on mRNA expression of A549 cells.** Downregulation of **(A)** Bcl-2 and upregulation of **(B)** Bax at gene expression level induced by AMEAE at 20 and 40 μg/mL concentrations after 24 h assessed by quantitative PCR analysis. The data represent the means ± SEM of three independent experiments. *p < 0.05 compared with the untreated group.

The mitochondria-initiated events are tightly regulated by the Bcl-2 family of proteins, including pro-apoptotic and anti-apoptotic proteins. Progression and abortion of apoptosis are governed by 25 genes in this family of proteins [40]. The pro-apoptotic protein of Bax is involved in the cytochrome *c* release from mitochondria to cytosol via dimerization and translocation to the outer mitochondrial membrane [41]. Meanwhile, anti-apoptotic proteins such as Bcl-2 suppress the translocation of cytochrome *c* (5). The levels of both Bcl-2 and Bax mRNA expression were estimated by using quantitative PCR analysis. The results demonstrated that Bcl-2 expression decreased significantly when A549 cells were treated with 20 and 40 μg/mL of AMEAE after 24 h as compared with the control cells (Figure 8). The expression of the proapoptotic Bax, however, increased significantly when treated with 20 and 40 μg/mL of AMEAE (Figure 8). Therefore, downregulation of Bcl-2 and upregulation of Bax upon AMEAE treatment could lead to loss of MMP, which facilitated cytochrome *c* release and activation of the caspase cascade. These changes in the gene expression of Bcl-2 and Bax confirmed the induction of apoptosis via mitochondrial-mediated intrinsic pathway.

### NF-κB translocation suppressed by AMEAE

**Figure 9:**
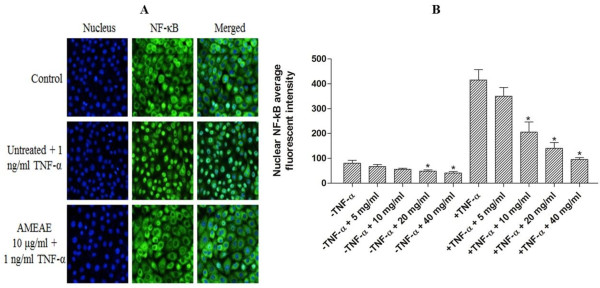
**Effect of AMEAE on NF-κB activation. (A)** Representative images of the intracellular targets of stained A549 cells that were exposed to AMEAE (10 μg/mL) and then stimulated with TNF-α. **(B)** Representative bar chart revealed the translocation of NF-κB nuclear in treated A549 cells with AMEAE. The data represent the means ± SEM of three independent experiments. *p < 0.05 compared with the untreated control group.

The ability of nuclear factor-κB (NF-κB) to inhibit the induction of apoptosis is considered to be involved in resistance against cytotoxic therapies. A variety of stimuli namely, anticancer agents and cellular stress can trigger NF-κB activation, which has been linked to extrinsic signaling pathway and inducible chemoresistance [42]. Thus, suppression of NF-κB translocation in conjunction with chemotherapy can effectively elevate the effect of cancer therapy [43]. In this study, the ArrayScan HSC system was used to determine the role of AMEAE in the suppression of activated NF-κB. In control cells, a high NF-κB fluorescence intensity was detected in the cytoplasm of A549 cells compared to the nuclei (Figure 9A). After treatment with AMEAE, nuclear NF-κB fluorescent intensity was dose-dependently reduced (Figure 9B). Stimulation of A549 cells by TNF-α resulted in a significant elevation in NF-κB fluorescence intensity in the nuclei, while, A549 cells treated with AMEAE elicited significant suppressive effects on the activation of NF-κB (Figure 9A and B), at 10, 20 and 40 μg/mL in a concentration-dependent manner. The present findings demonstrated that AMEAE treatment can effectively reduce the activation of NF-κB signaling pathway in A549 cells. We have shown here that the NF-kB translocation can be suppressed by AMEAE, which suggests the involvement of an NF-κB inhibition mechanism in apoptosis.

## Conclusions

In conclusion, the anticancer potential of ethyl acetate extract of *A. muricata* leaves was supported by the evidence provided in the present study, including lactate dehydrogenase leakage, reactive oxygen species generation, loss in mitochondrial membrane potential, increase in the level of cytochrome *c*, upregulation of Bax, downregulation of Bcl-2 and activation of initiator and executioner caspases. The antiproliferative effect of AMEAE was accompanied by cell cycle arrest at G_1_ phase and suppression of NF-κB translocation. The results confirmed the involvement of intrinsic pathways in induced apoptosis.

## Abbreviations

AMEAE: *A. muricata* leaves ethyl acetate extract
HCS: High content screening
NF-κB: Nuclear factor-kappa B
MMP: Mitochondrial membrane potential
LDH: Lactate dehydrogenase
ROS: Reactive oxygen species (ROS)
Bax: Bcl-2 associated X protein
Bcl-2: B-cell lymphoma protein 2
DHE: Dihydroethidium.
